# Telemetry reveals rapid duel-driven canary song plasticity in a competitive social environment

**DOI:** 10.3389/fpsyg.2024.1468782

**Published:** 2025-03-05

**Authors:** Pepe Alcami, Shouwen Ma, Manfred Gahr

**Affiliations:** ^1^Department of Behavioural Neurobiology, Max Planck Institute for Biological Intelligence, Seewiesen, Germany; ^2^Division of Neurobiology, Faculty of Biology, LMU Munich, Martinsried, Germany

**Keywords:** birdsong, duel, countersinging, behavioral plasticity, behavioral flexibility, fine motor skill, social behavior, songbird

## Abstract

Singing by songbirds is a sexually selected, complex motor skill that is learned during juvenile development. In open-ended learners, adult songs are plastic, that is, birds retain the ability to change their songs. In some seasonal open-ended learners, including canaries, songs become stable at the onset of each breeding season. However, whether context-dependent plasticity of songs occurs during the breeding season remains elusive. We used custom-made telemetric backpack sound recording technology in five groups of canaries to monitor song-based communication from three males in competition for females during the breeding season. This allowed us to record each male’s songs during social interactions. We show that canaries proactively overlap their songs in time during aggressive vocal exchanges that we call duels. Birds that engage in duels take leader or follower roles on a song-to-song basis. When a male canary leads a duel, his songs last longer relative to his solo songs, increasing the chance to outlast the follower’s song. Moreover, the durations of leader and follower songs in duels are correlated, suggesting an interactive online adjustment of their songs. Remarkably, in each group, only two out of the three males extensively engage in duels whereas the third canary rarely participates. Overall, our findings reveal context-dependent behavioral flexibility of male-directed canary song signaling, characterized by a moment-to-moment plasticity different from the slow, well-studied seasonal plasticity. By their context-dependent modulation of the relative timing and duration of vocal exchanges, canary duels offer a window into the social cognitive abilities of songbirds.

## Introduction

The social context in which the behavior of individual animals takes place is a key component of natural environments in which animals have evolved (Svensson and Sheldon, 1998). Social networks influence the production and fine-tuning of learned motor skills, such as vocal communication signals (McGregor et al., 1993; King et al., 2013; Ota et al., 2018). Singing by songbirds is a complex learned motor skill influenced by conspecifics and involved in a variety of social functions, including mate attraction, pair bonding, parental investment, territorial behavior, and signaling of social hierarchy (Kroodsma, 1976; Ficken et al., 1978; Sossinka and Böhner, 1980; Payne, 1983; King and West, 1989; Tchernichovski et al., 1999; Beecher et al., 2000; Collins, 2004; Garcia-Fernandez et al., 2010; Akçay et al., 2013). In many songbird species, individuals engage in interactive singing, known as countersinging, or dueling when it occurs between rivals (Todt and Naguib, 2000; Logue, 2021). During countersinging, the timing and acoustic properties of songs can encode aggressive information, particularly when songs overlap in time (Dabelsteen et al., 1997; Mennill and Ratcliffe, 2004; Naguib and Kipper, 2006; Vehrencamp, 2001; Searcy and Beecher, 2009; Naguib and Mennill, 2010). However, little is known about whether songs are plastic, that is, whether, and to what extent, they change their properties, specifically in response to conspecific songs.

Despite male canaries actively overlapping playbacks of acoustic noise with their songs, resulting in plasticity of song amplitude (Goto et al., 2023; Hardman et al., 2017), whether canary songs trigger overlaps and plasticity of conspecific songs remains unknown. Interestingly, the preference of female canaries for temporally overlapping songs has been well characterized. In particular, females prefer playbacks of “overlapping” songs (song playback starting last during the interaction) to playbacks of “overlapped” songs (Leboucher and Pallot, 2004). Furthermore, female canaries allocate more yolk in their eggs in response to overlapping songs, highlighting the importance of singing overlaps for biological fitness (Garcia-Fernandez et al., 2010). Although canaries are a model species for studying both vocal plasticity and the behavioral and ecological impact of singing overlaps on females, whether overlaps occur during the breeding season in spontaneously interacting canaries and whether male canaries adjust their songs, specifically their duration, depending on the interaction between males remains elusive. Song duration is a particularly relevant property, being testosterone-sensitive, contributing to female preference (Kroodsma, 1976; Heid et al., 1985) and remaining variable during the breeding season (Markowitz et al., 2013), while other song features crystallize (Alliende et al., 2017).

The majority of studies on countersinging have used song playbacks, which lack the essential interactive component between singing birds. The study of directly interacting birds has been hindered by the need to disambiguate signals from individual birds, particularly when songs overlap in time. One approach to overcome this difficulty involves the use of microphone arrays (Araya-Salas et al., 2017), but they can lack the temporal and acoustic precision to characterize singing interactions in detail due to the distance from sound sources. An alternative solution is the use of individual microphones mounted on each bird (Gill et al., 2016). In this study, using “backpack microphones” in aviaries, we confirmed the temporal overlaps observed in the field and characterized the relative singing dynamics of male canaries and their impact on song duration.

## Results

### Multiple-microphone recordings in the field reveal temporally overlapping songs

**Figure 1:**
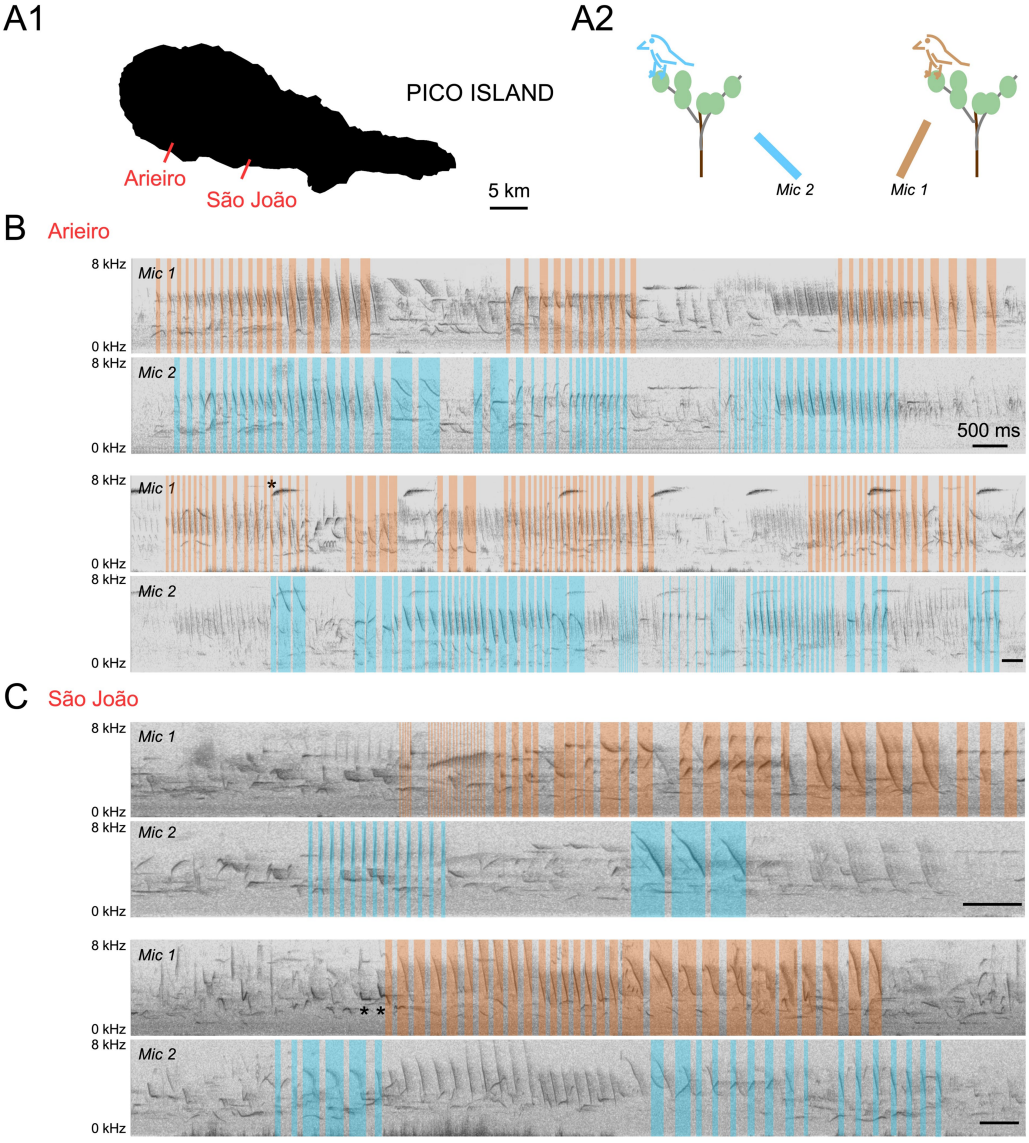
Multiple microphone recordings reveal overlapping songs from wild canaries. **(A1)** Recording sites are indicated on a map of Pico Island. **(A2)** Recording configuration. Two directional microphones were positioned to capture sounds from different directions in a field where canaries had been located. **(B)** Spectrograms of two example overlapping singing periods in Arieiro. **(C)** Spectrograms of two example overlapping singing periods in São João. Syllables from putative canary songs from two different canaries recorded preferentially by microphone 1 (Mic1) or microphone 2 (Mic2) are labeled in orange and blue, respectively. Scale bars, 500 ms. Note that the recordings also capture additional distant canary songs (top) and the vocal production of additional species (examples are annotated with *).

We first asked whether canaries overlap their songs in their natural environment. For this purpose, we recorded wild canaries during the breeding season in Pico island (Azores, Portugal) using two directional microphones oriented toward different directions in fields where canaries had been located. Sound spectrograms revealed canary song temporal overlaps, as shown by overlapping songs from different canaries being preferentially recorded by one or the other microphone (Figure 1).

In the same fields where canaries were recorded, several other bird species vocalized and sometimes overlapped canary songs. Frequently recorded species include chaffinches (*Fringilla coelebs* *moreletti*), blackbirds (*Turdus merula* *azorensis*), greenfinches (*Chloris chloris*), blackcaps (*Sylvia atricapilla*), and robins (*Erithacus rubecula*). The complexity of vocal exchanges performed by multiple species simultaneously in the field precluded further analysis of spontaneous singing dynamics.

### Telemetric recordings in naturalistic social environments reveal aggressive singing duels

**Figure 2:**
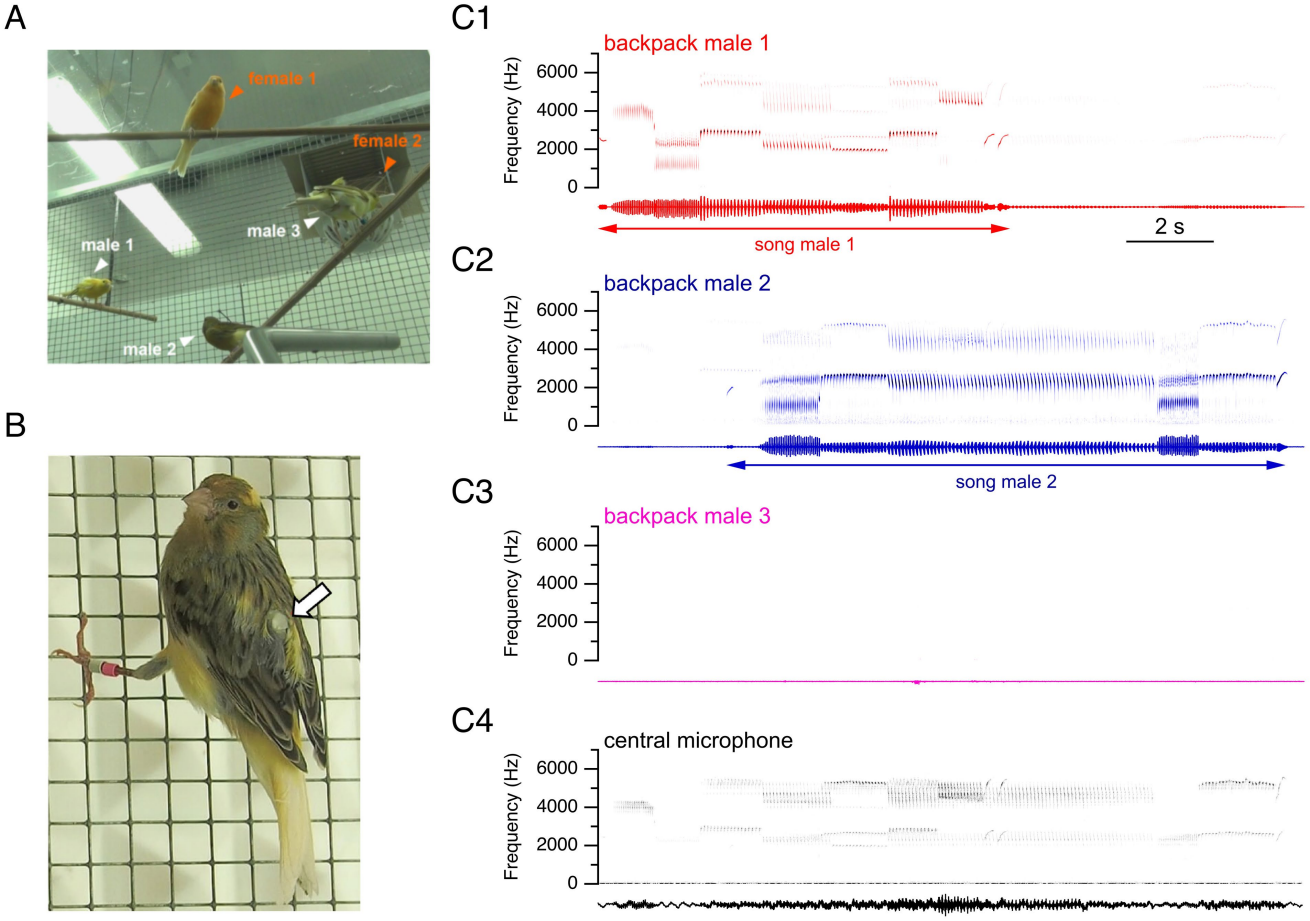
Telemetric recordings with backpack microphones make it possible to disambiguate individual birds’ songs during temporally overlapping singing periods. **(A)** Experimental design: mixed sex groups comprising two female and three male canaries, with all three males individually equipped with backpack microphones. Note that female 2 is in the nest behind male 3. **(B)** Male canary equipped with a backpack microphone, indicated by the arrow. **(C)** Representative simultaneous recordings from three backpack microphones **(C1–C3)**, each worn by a different male canary, and a central microphone **(C4)** that records vocalizations from all birds in the room. Top: sonograms. Bottom: sound waveforms. The recordings show simultaneous vocalizations captured by backpack microphones during an overlapping interaction, lines with arrows indicating the identity of the singing bird, and the start and end of songs. Attenuated sounds from other birds’ songs can also be recorded at lower amplitudes on backpack microphones.

To further characterize the intraspecific singing dynamics of male canaries with improved temporal detail, and in controlled social environments that exclude interspecific interactions, we designed a social experimental setting in aviaries. Specifically, we housed mixed groups of three males and two females, reproducing sex ratios previously reported in the wild (Voigt and Leitner, 1998) and establishing a context in which males compete for females. We equipped each male with custom-made backpack microphones (Gill et al., 2016) monitored with the help of a telemetric recording system (Figures 2A,B). Backpack microphones enabled us to unambiguously identify each singing male from the simultaneous recordings of males in the group (Figures 2C1–C4).

**Figure 3:**
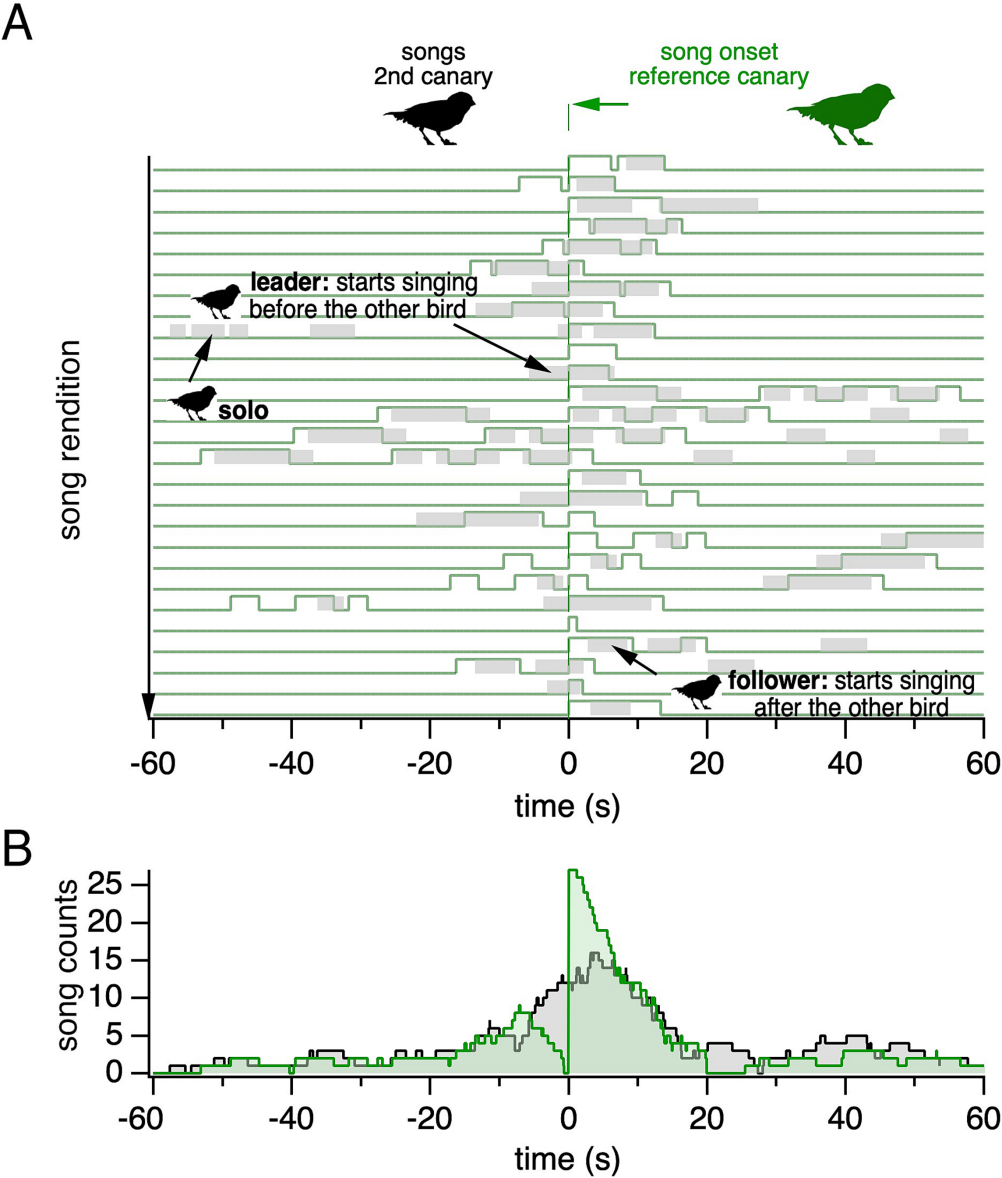
Songs sung by backpacked canaries reveal solo and temporally overlapping songs. **(A)** Song onsets from the reference canary (green) are aligned at time = 0 s over a 4-h recording period. Song renditions are plotted along the Y-axis. Songs from a second canary (gray) are shown relative to the aligned song onsets of the reference canary. **(B)** The corresponding song histogram shows the summed song counts over time for the second canary (black). The reference canary’s song histogram is also shown (green).

To visualize the temporal relationship between songs sung by two canaries, we plotted the songs of one canary relative to the onsets of another canary’s songs. This representation, including the reference canary’s songs, highlights that both canaries sing solo and temporally overlapping songs (Figure 3A). Note that in this article, the term “overlapping” refers to songs that overlap in time, irrespective of the temporal order of songs within overlaps. During overlaps, a canary may lead by starting to sing before the other bird; or follow by starting after the other bird. The two canaries can switch roles on a song-to-song basis. The corresponding histogram of a canary’s songs, plotted relative to a reference canary’s song onset, reveals a peak of singing activity at a short interval relative to the reference canary’s song onset (Figure 3B). The histogram shows two timescales: a short timescale of several seconds, primarily reflecting songs overlapping each other in time, and a longer timescale of tens of seconds, resulting from song bouts.

**Figure 4:**
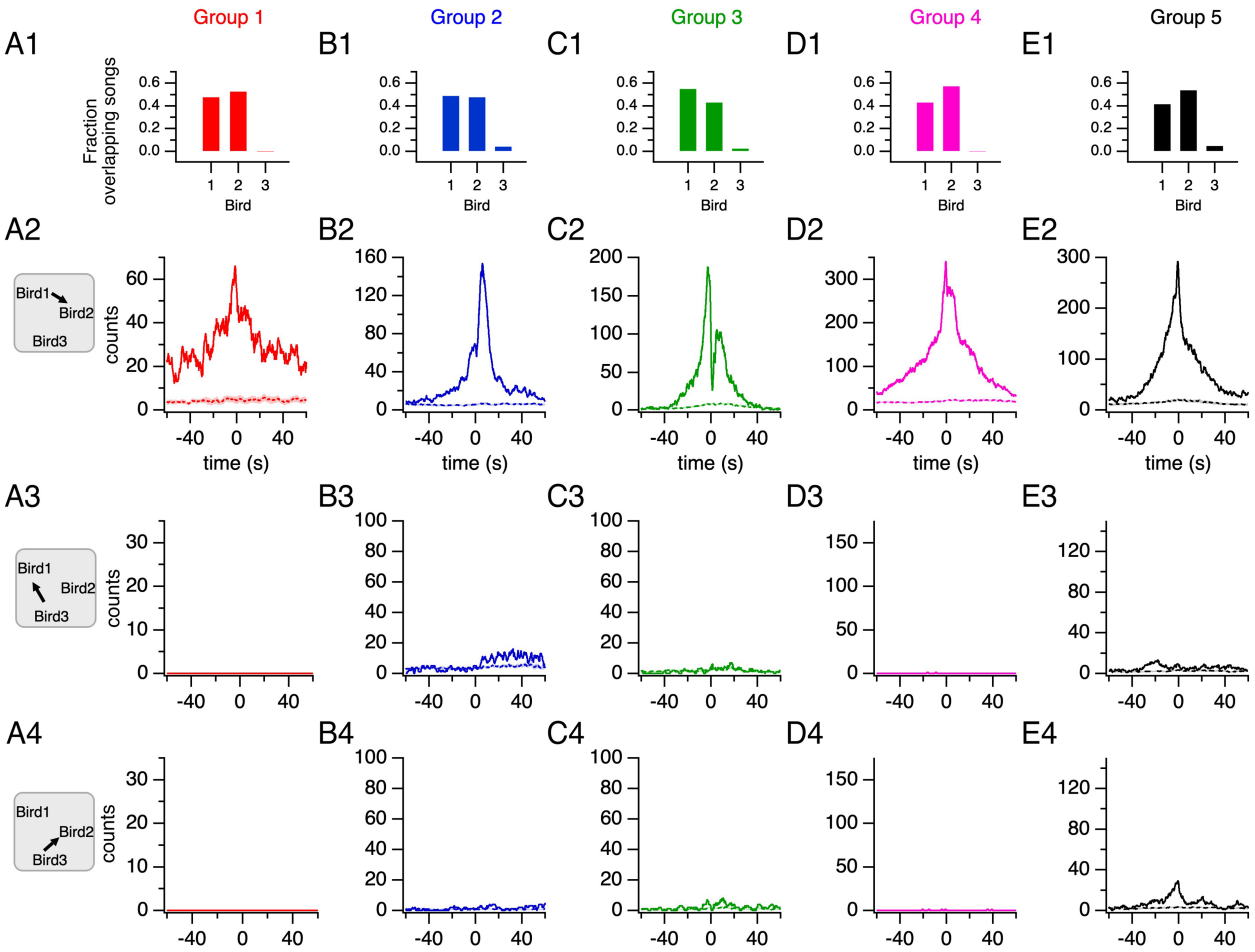
Two main duelists in each group time their song production above chance levels. **(A1–E1)** Fraction of total overlapping songs contributed by each bird in each group. The two main duelists in each group (birds 1 and 2) contribute the majority of overlapping songs. **(A2–E2)** Song histograms of bird 1 relative to bird 2 show increased singing activity for one of the main duelists within 60 s of the other’s song onset (solid lines). Histograms generated from shuffled songs (dashed lines: average ± 95% confidence interval) confirm that the observed distributions reflect increased singing within the 60-s time window relative to random overlaps. **(A3–E3)** Song histograms for bird 3 aligned to bird 1’s song onsets. **(A4–E4)** Song histograms of bird 3 aligned to bird 2’s song onsets.

In all groups, two male canaries each contributed at least 40% of the group’s overlapping songs whereas the third canary contributed less than 5% (Figures 4A1–E1; Supplementary Table S1). Thus, singing by canaries in this competitive setting is characterized by two different behavioral phenotypes: two frequent duelists and a third bird rarely engaging in duels.

The histogram of songs recorded from one of the main duelists plotted relative to the song onsets of the other duelist reveals an increased singing activity within 60 s of song onset from the reference bird (Figures 4A2–E2). The comparison with song histograms generated from shuffled songs reveals that singing overlaps are not random, but instead, a proactive behavior. The histograms for the third bird in each group relative to each of the two main duelists were also generated (Figures 4A3–E3,A4–E4, respectively). Alternatively, the corresponding histograms of song onsets, representing only the times at which songs are initiated, revealed the distribution and the variability of song onset time from a duelist relative to another duelist’s song onset across duelists (Supplementary Figure S1).

Finally, to investigate the aggressive signaling nature of canary song overlaps, we quantified the relationship between singing and physical fights from video recordings. Song temporal overlaps were followed by fights in 52 ± 12% of the cases on average, compared to 13 ± 6% for solo songs (*n* = 3 groups, Fisher’s exact test; *p* < 0.05; Supplementary Table S2). Thus, song overlaps are aggressive signals predictive of physical fights, justifying the term “duel.”

### Dynamics of leader and follower roles across days

**Figure 5:**
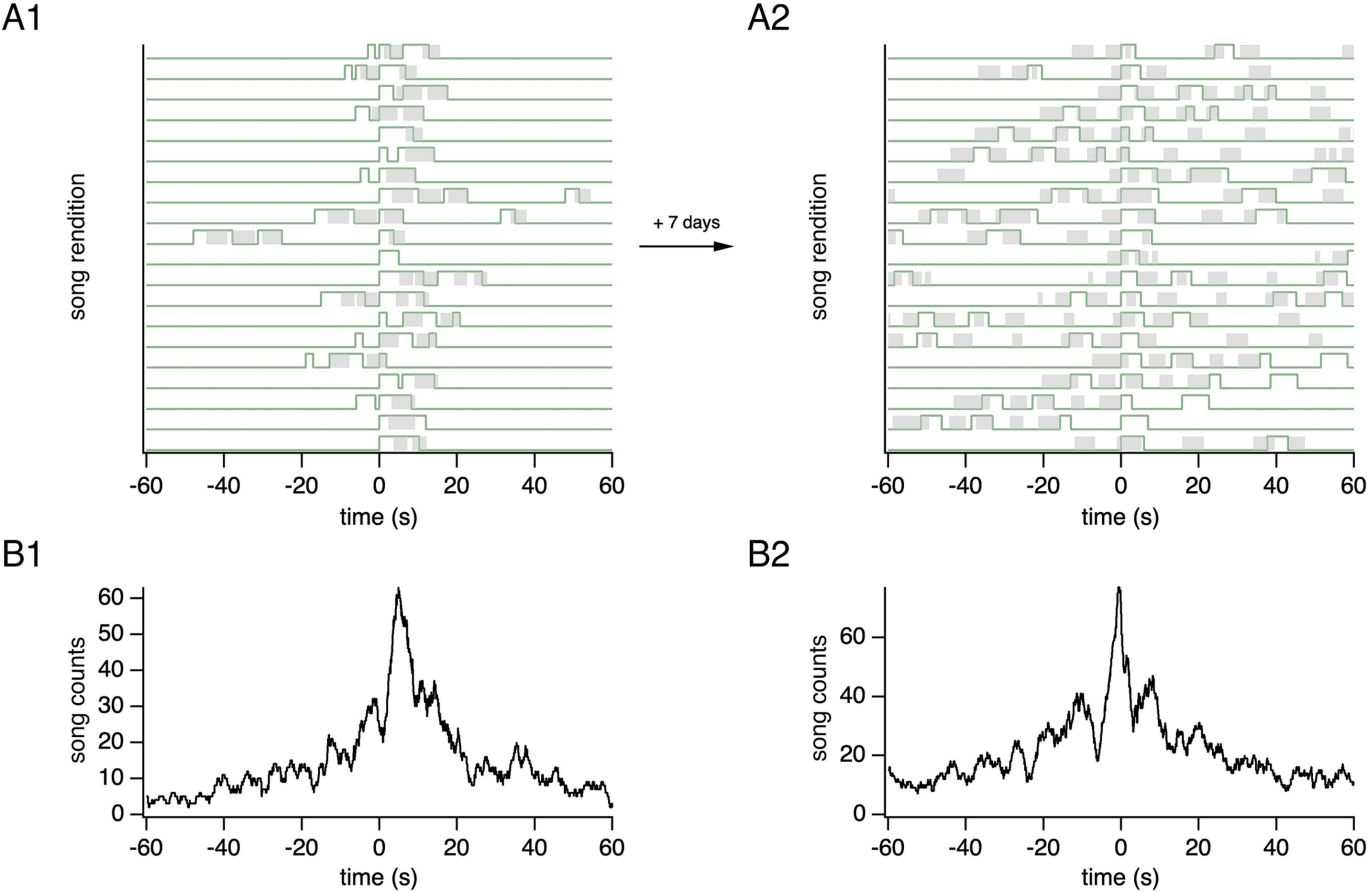
Temporally evolving follower and leader roles across days. **(A1)** Twenty representative consecutive song alignments from a pair of duelists recorded during 1 day. The songs of both birds 1 and 2 (gray and green) are shown relative to song onset from the reference bird 2 (green), aligned at time = 0 s. **(A2)** Twenty representative consecutive song alignments from the same birds recorded after 7 days. **(B1)** Song histograms corresponding to all songs sung by bird 1 on the day shown in **(A1)**. The histogram increases at positive values, reaching a peak that mostly reflects songs from the bird acting as follower. **(B2)** Song histograms corresponding to all songs sung by bird 1 on the day shown in **(A2)**. The portion of the song histogram that increases at negative values before the song onsets of the reference bird’s songs (time = 0 s) mostly corresponds to leader songs. Note the change in the main leader and follower roles of birds between the 2 days: The bird represented in black shifts from a predominantly follower role in **(A1,B1)** to a predominantly leader role in **(A2,B2)**. Conversely, the reference bird (green) transitions from predominantly leading in **(A1,B1)** to predominantly following in **(A2,B2)**.

Songs were recorded for days, during which the two main duelists in each group performed solo songs, leading songs or following songs on a song-to-song basis. Leader/follower dynamics can vary over time. The analysis of two males in a group indicated a reversal of their relative predominant leader/follower roles after several days (Figure 5).

### Leading, following, and solo songs differ in duration

We next examined the influence of the social singing context on songs on a song-to-song basis. To this end, we quantified song duration in different overlapping conditions. A total of 3,629 solo songs and 2,629 temporally overlapping songs were recorded from 15 males (3 birds per group, 5 groups, Supplementary Table S1).

We fitted a linear mixed-effects model with the logarithm of song duration as the response variable, song type (“Solo,” “Leader,” “Follower,” and “Following a song and then leading the next song”) as the independent categorical variable, and bird identity as a random effect. The logarithm of song duration is predicted by song type. Solo songs were estimated to last 3.834 s. Leader songs have an estimated duration of 5.942 s, significantly longer than solo songs (*t* = 19.868, *p <* 2 × 10^−16^), whereas follower songs are estimated to last 3.384 s, less than solo songs (*t* = −6.098, *p* = 1.14 × 10^−9^). Songs that start following a song but then lead the next song lasted 6.214 s, making them the longest song type relative to solo songs (*t* = 13.407, *p <* 2 × 10^−16^).

**Figure 6:**
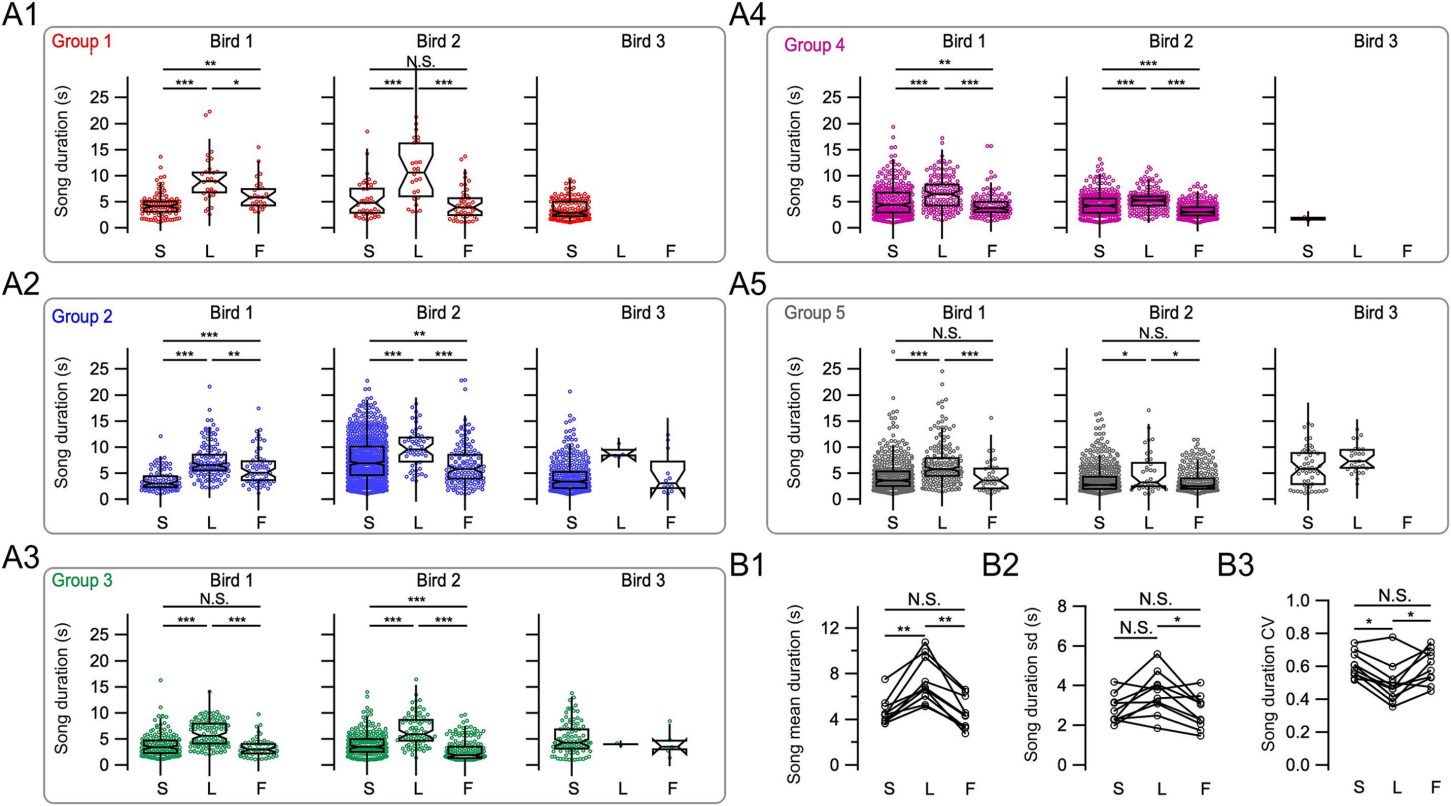
Male canaries song duration and its variability depend on the relative timing of song onset. **(A1–A5)** Song duration for the three male canaries in each of the five groups was plotted, corresponding to groups 1–5 shown in Figure 4. The three different birds in a group are shown in the left, middle, and right panels. S, solo songs; L, leading songs; F, following songs. Multi-comparison Dunn’s tests were performed for the two main duelists in each group, *p < 0.05, **p < 0.01, ***p < 0.001, and N.S. *p* > 0.05. **(B1)** Summary data show differences in average song duration for solo, leader, and follower songs for 10 birds in five groups. **(B2)** Summary data for the standard deviation of song duration for solo, leader, and follower songs. **(B3)** Summary data for the coefficient of variation of song duration (CV) for solo, leader, and follower songs. In **(B1–B3)**, open symbols represent the values from each individual bird, and values from the same bird for different song types are linked (S, solo songs; L, leading songs; F, following songs). *p < 0.05, **p < 0.01, N.S. *p* > 0.05 two-tail Wilcoxon signed-rank test with Bonferroni-Holm correction.

To investigate the impact of clearly defined leader or follower roles on song duration, we excluded from the following analysis songs that could be categorized as both following songs in a first interaction and leading songs in a second successive interaction or complex leader–follower interactions between the three birds (*n* = 327 songs). We tested for each duelist separately whether their songs differed across the three song categories: solo, leader, and follower. In all canaries, leader songs were significantly longer than both solo and follower songs (Kruskal–Wallis test followed by Dunn’s test with Bonferroni-Holm correction, *p <* 0.05, Figures 6A1–A5; Supplementary Table S3). However, follower songs showed variable trends relative to solo songs when examined for each individual bird: They increased in two canaries, decreased in four, and did not change in another four, relative to solo songs. These results further highlight the interindividual variability in canary dueling.

On average, leading song duration for the main two duelists of all groups was ~59% longer than solo songs (7.38 ± 0.62 s vs. 4.63 ± 0.37 s, two-tail Wilcoxon signed-rank test, Bonferroni-Holm corrected: *p* = 0.0059, *n* = 10 canaries, Figures 6B1) and ~ 64% longer than following songs (4.50 ± 0.44 s, p = 0.0059). Average solo song and follower song durations did not differ across all duelists (*p* = 0.43).

We further examined whether the variability of song duration was changing during singing interactions (Figures 6B2). The standard deviation of leader song duration did not significantly differ from solo songs (3.61 ± 0.34 s vs. 2.79 ± 0.23 s, respectively, *p* = 0.0742, two-tail Wilcoxon Signed-Rank test, Bonferroni-Holm corrected), and the standard deviation of leading songs was ~ 35% longer than following songs (2.67 ± 0.27 s, *p* = 0.0293). The standard deviation of solo and following song duration was not significantly different (*p* = 0.323). We also examined the coefficient of variation of song duration as an additional measure of variability (Figures 6B3). Interestingly, the coefficient of variation of song duration decreased by ~ 18% for leading songs relative to solo and follower songs (0.49 ± 0.04 vs. 0.61 ± 0.02 and 0.60 ± 0.03, respectively, two-tail Wilcoxon signed-rank test, Bonferroni-Holm corrected: *p* = 0.0176, and *p* = 0.0391), and did not differ between solo and follower songs (*p* = 0.625).

In summary, song duration and its variability depend on the relative timing in the singing interactions.

### Longer-duration songs increase the likelihood that leaders outlast followers

**Figure 7:**
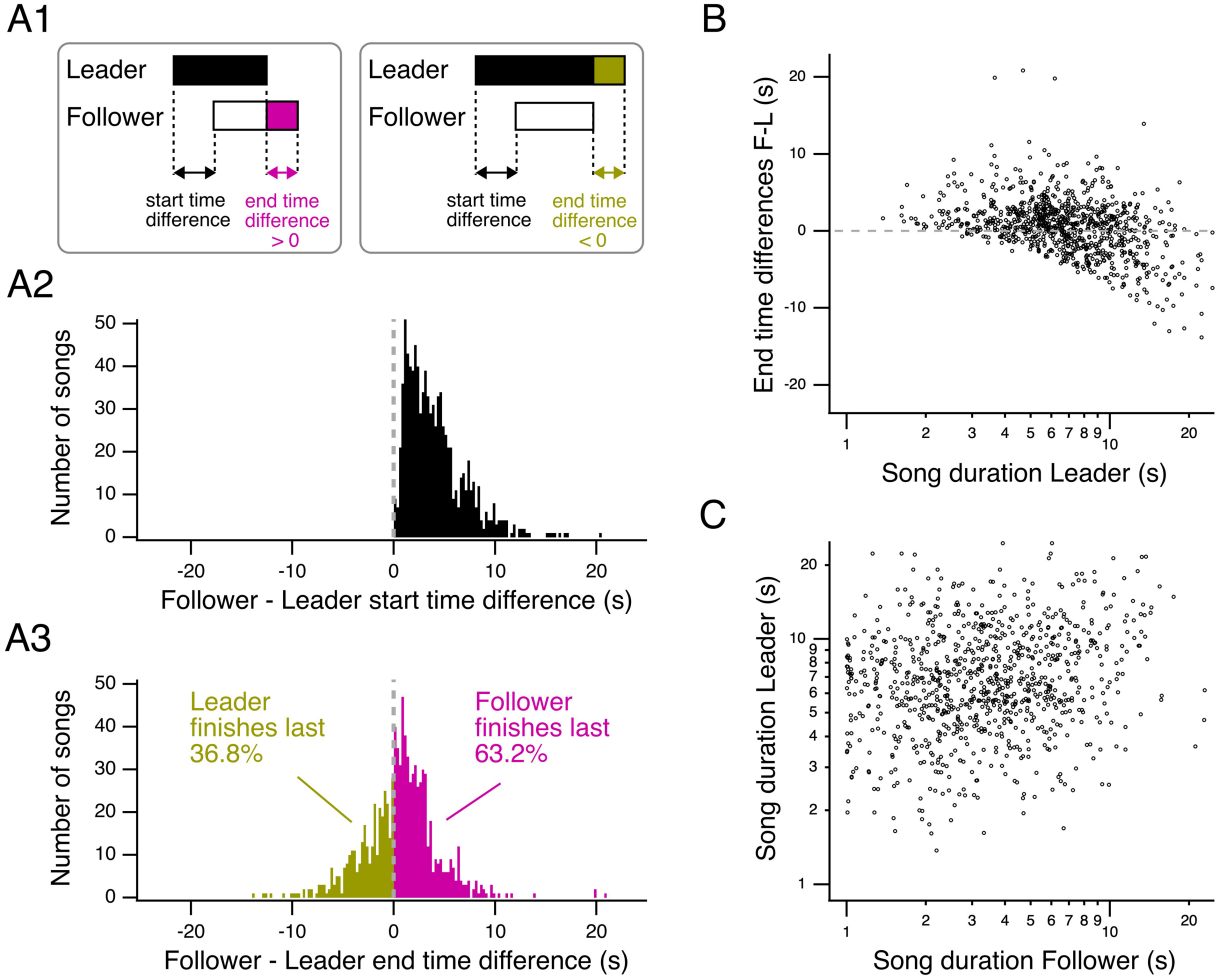
Relationship between leader and follower songs: start and end time differences and song duration. **(A1)** Diagram representing overlapping songs and the terminology used. Left, when leaders are outlasted by followers, the end time difference is positive. Right, when leaders outlast followers, the end time difference is negative. **(A2)** Histogram representing the start time differences for all interactions with clearly defined leader or follower songs for the two main duelists of all groups. **(A3)** Histogram representing the corresponding end time differences for the two main duelists of all groups. **(B)** End time differences as a function of leader song duration show a negative correlation for the two main duelists of all groups (linear mixed-effects model *p <* 2 × 10^−16^). **(C)** Leader and follower song duration are positively correlated for the two main duelists of all groups (linear mixed-effects model *p* = 7.52 × 10^−6^). Gray dashed lines indicate end-time differences of 0s. Leader and follower song durations are plotted on a logarithmic axis in **(B)** and **(C)**.

Follower songs, starting last in a duel, have an advantage in outlasting the opponent’s song (Figures 7A1,A2). We hypothesized that the ability of a leading song to outlast a following song may be facilitated by longer leading songs. To test this hypothesis, we first examined the difference in end times between following and leading songs for the main duelists of all groups (Figure 7A3). End time differences follow a unimodal distribution, with negative and positive values, peaking at the 0.75–1 s time bin. Thus, canaries tend to end their songs within short time intervals relative to each other, with a mode at positive values. Follower songs outlasted leader songs in ~63% of cases, while leader songs outlasted follower songs in ~37% (Figure 7A3). Therefore, although leaders sang longer songs on average, follower songs were more likely than leader songs to outlast the opponents’ songs.

We then plotted the end time differences as a function of leader song duration (Figure 7B). We fitted a linear mixed-effects model to test the relationship between the end time differences between follower and leader songs (response variable) and the logarithm of leader song duration (independent variable), while accounting for random effects (bird identity and group). The model indicates that end time differences are predicted by the logarithm of leader song duration (intercept = 6.2602, slope = −6.6574 ± 0.5032 (SE), *t* = −13.23, *p <* 2 × 10^−16^). The model shows a significant negative relationship between end time differences and leader song duration: longer leader song durations are associated with more negative end time differences. In other words, as the leader song duration increases, leaders outlast followers to a greater extent.

### Leader and follower song durations are correlated

Finally, we examined whether leader and follower song duration are correlated across leader–follower song pairs from the main duelists (Figure 7C). We fitted a linear mixed-effects model with the logarithm of leader song duration as the response variable, the logarithm of follower song duration as the independent variable, and group and leader identity as random effects. The model confirmed that longer follower song durations are significantly associated with longer leader song durations (intercept = 0.79099, slope = 0.12359 ± 0.02743 (SE), *t* = 4.505, *p = 7.52 × 10^−6^*). Thus, birds adjust their song duration relative to each other in leader–follower interactions, supporting the hypothesis of online, moment-to-moment adjustments in dueling songs.

## Discussion

### Socially induced song plasticity

We found that male canaries overlap their songs during the breeding season and that these are accompanied by aggression. Canary duels are characterized by context-dependent plasticity in which songs become longer when birds lead the duels. This result adds to a body of evidence suggesting that songs retain a degree of plasticity even after they have crystallized (Tumer and Brainard, 2007; Leonardo and Konishi, 1999). Remarkably, plasticity in song duration takes place at much faster timescales than the slower, well-studied song seasonal plasticity (Leitner et al., 2001; Nottebohm et al., 1986; Alliende et al., 2017). Future analysis of the fine structure of songs at a shorter timescale, focusing on syllables, should provide further insights into the extent of plasticity taking place during duels.

During song competitions, songbirds might take turns to avoid overlap (Hultsch and Todt, 1982; Pika et al., 2018) or adjust their songs to avoid jamming. This might be achieved, for example, by increasing the amplitude of the signal (Hardman et al., 2017), by using song types that differ from those of the competitor (Vehrencamp, 2001), or by shifting pitch (Tumer and Brainard, 2007). Nevertheless, female perception of individual songs during temporal overlaps may be difficult. When male canaries outcompete their rivals by prolonging their songs, the identity of the bird finishing last and the ending part of his song may be well perceived by the female.

An important consideration that might influence the interpretation of our results is the controlled number of canaries per group studied with telemetry in an aviary. Although this setting made it possible to quantify plasticity during singing interactions in small groups of domesticated canaries and reproduce a competitive environment with sex ratios similar to those in the wild, it still does not fully correspond to the social environment found in their natural habitat (Voigt and Leitner, 1998). Recording with telemetry from groups of wild canaries in the field poses major challenges that should be addressed in the future to further characterize the singing behavior of canaries.

### Neurobiological implications

The context-dependent plasticity of canary songs suggests a socially induced differential activation of the premotor networks that underlie song production. In this regard, the recording of song-control brain nuclei during canary duels will be of great interest to understanding how the activity of birdsong premotor networks is reconfigured in social environments. Songs from other canaries and not only the bird’s own song (Lehongre and Del Negro, 2009) have been shown to evoke auditory responses in the nucleus HVC, a pallial region that encodes timing during singing (Hahnloser et al., 2002; Liberti et al., 2016). HVC recordings during duels should elucidate how the song of overlapping conspecifics influences ongoing premotor neural activity.

We find that the relative timing of singing influences song properties on a song-to-song basis, suggesting that acoustic information and social cues feed into ongoing motor commands controlling singing. A powerful inhibitory gating of HVC auditory responses has been reported during singing (McCasland and Konishi, 1981; Schmidt and Konishi, 1998). It would therefore be interesting to further investigate whether inhibitory gating of auditory responses in HVC is released during singing duels. Remarkably, neurons coding for song history for up to several seconds have been described in the premotor nucleus HVC from canaries (Cohen et al., 2020). These neurons have been proposed to constitute hidden neural states that organize songs at the timescale of seconds. When a canary faces an overlap with another canary, longer-lasting song segments occurring during the remainder of the plastic song may be encoded by such cells.

Furthermore, the nucleus LMAN of the anterior forebrain pathway of the song control system may also be involved in the fast song plasticity of canary songs described here. This area is known to generate variability in the song system (Alliende et al., 2017). In relation, the firing pattern of LMAN changes depending on the social context during which singing takes place in zebra finches and correlates with small modifications of the uttered song (Kao et al., 2008).

### Ecological and evolutionary implications

Female canaries perform more frequent copulation solicitation displays when they are presented with “overlapping” songs relative to “overlapped” songs (Leboucher and Pallot, 2004; Amy et al., 2008). Thus, female preference for overlapping songs may have exerted an evolutionary pressure on males to sing longer-lasting leading songs to outlast a follower song, if what females prefer is which bird outlasts the opponent in the duel. Whether acoustic matching and specific syllables take place during duels as well as their impact on females should be further characterized. Indeed, female canaries prefer “overlapping” to “overlapped” songs, but this preference disappears if “sexy” syllables are presented (Leboucher and Pallot, 2004). Likewise, female preference may have selected over evolutionary timescales the neural mechanisms allowing canaries to integrate social contextual information during duels and sing longer-lasting songs.

Overlapping songs have been studied previously in a context of constant song duration, where leader playbacks start and end first, and follower playbacks start and end last. However, leaders can outlast followers and increase the chance to outlast opponents in a duel by increasing song duration, thereby introducing an additional dimension to temporal overlaps. Whether females prefer the bird that follows, and/or the bird that outlasts, requires further investigation. The preference of females for solo and overlapping songs should therefore be studied in interactive environments in the future, taking into account this additional temporal dimension present in overlapping interactions.

Remarkably, dueling male canaries are able to increase song duration by ~60%, which means to utter ~60% more motor gestures (syllables) during the continuing performance of a leading song. Likely, only males in excellent physical condition are able to do so. Long songs are sung only by canaries that have been exposed to elevated levels of testosterone for longer periods, which is a sign of fitness (Heid et al., 1985; Gil and Gahr, 2002). We therefore propose that canary duels constitute honest signals, as they are associated with longer-lasting songs, and they are an honest predictor of the willingness or ability to fight. Moreover, the capacity of a bird to duel indicates sensorimotor coordination abilities. Note that although the duelists’ ability to sing longer may be related to testosterone and build up during the progression of duels, it is not known whether individual differences in dueling abilities can be traced back to individual differences in testosterone level in a competition context.

The fact that two canaries perform the majority of duels while the third canary rarely participates may be related to differences in testosterone levels, singing abilities, and social hierarchy. Alternatively, it may be related to a mismatch of male and female numbers, a situation in which the two main duelists may compete for the same female, whereas the third bird may be paired with the other female, possibly pointing to different mating strategies.

How duels impact fitness is unclear at this stage. Indeed, although females prefer overlapping singers, they also show a preference for canaries that do not participate in physical contests (Amy et al., 2008). Whether fitness may be related to dueling abilities should further be investigated in wild populations (Bircher et al., 2023). Since in our experimental conditions, singing duels were often accompanied by physical aggression, future experiments in the field should reveal whether physical aggression also occurs in natural conditions between dueling birds, and the biological fitness associated with different singing strategies: dueling as a leader, as a follower, or singing solo songs.

Overlapping songs have been proposed to constitute signals (Searcy and Beecher, 2009; Naguib and Mennill, 2010). Our results support that canary duels are a signal: temporal overlaps occur in spontaneous temporally overlapping interactions above chance levels, they are predictive of increased aggression, and only a subset of canaries in a group overlap. Moreover, songs change during overlaps, suggesting their signaling content may also change. Our study further raises the question of how singing duels may have shaped the evolution of song culture in canary populations.

In this article, we show that adult canaries perform complex interactive duels, inducing context-dependent song plasticity. Interestingly, birdsong has been proposed to be a cognitive ability (Searcy and Nowicki, 2019). One could draw an analogy of canary duels with human vocal interactions, as canaries perform solo vocalizations as well as complex overlapping interactions during which vocalizations change in a context-dependent manner. We argue that the complex sensorimotor loop underlying the online modification of songs during duels involves the processing of complex information from rivals to modify ongoing signals, qualifying as a cognitive ability.

## Materials and methods

### Ethics permits

Bird housing and all experimental procedures, including the use of audio transmitters, were approved by the Government of Upper Bavaria (Ethical approval ROB-55.2Vet-2532.Vet_02–17-211) and performed according to the Directive 2010/63/EU of the European Parliament and Council of 22 September 2010 on the protection of animals used for scientific purposes.

### Animals and housing conditions

A total of 30 adult domesticated canaries (six groups formed by three males and two females each) were part of this study. Animals were randomly selected from larger aviaries and moved together in 2 m*1 m*1 m indoor aviaries or alternatively 1.68 m*0.78 m*1.68 m boxes. In both cases, birds were not within hearing range of other birds. Birds were kept in breeding conditions under a 13/11-h light/dark cycle (fluorescent lamps), at 24°C and 60–70% humidity. Food (mixed seeds, and “egg food”), fresh water, and cuttlebone were provided *ad libitum*, as well as nesting material and nests.

### Audio recordings on Pico Island

Two Sennheiser MKH 70 P48 directional microphones pointed in different orientations in a field where canaries had been observed. Audio recordings were made using a Marantz PMD661 recorder with an audio bit depth of 24 bit per sample. The recording areas (Figure 1) were located in Arieiro (38°26′29.5”N 28°28′40.6”W) and São João (38°25′10.9”N 28°20′05.0”W).

### Audio recordings at the Max Planck Institute for Ornithology

Custom-made wireless backpack microphones (0.6 g, including battery) (Gill et al., 2016) were mounted on the back of canaries and fixed with an elastic band around the upper thighs. The frequency-modulated radio signals were detected by AR8600 communication receivers (AOR, Ltd., Japan). Audio signals were fed into an eight-channel audio A/D converter (Fast Track Ultra 8R, Avid Technology, Inc., United States) and recorded with custom-made software. We additionally installed a central microphone (Earthworks TC20), also connected to the A/D converter, on top of the aviary. The sampling rate of sound recordings was 22,050 Hz.

### Video recordings

Videos for behavioral quantifications were recorded in aviaries using a CX405 handycamcorder (Sony) with built-in microphones.

### Song analysis

The analysis was performed using Audacity (version 3.1.3), MATLAB (version 2024.b), and Igor Pro (version 8.04). The onset, duration, and offset of songs were annotated manually on Audacity (version 3.1.3) by inspecting visually the sonograms from the three backpack recordings and the central microphone capturing all vocalizations in the room. Songs were defined by a minimal duration of 1s and did not contain silent intervals longer than 0.5s. Annotations were based primarily on the intensity of syllables recorded by different microphones and secondarily on relative timing. When channels were noisy and it was not possible to identify the singing bird combining the remaining recorded channels and the central microphone, songs were not annotated in the corresponding recording period, and these recordings were excluded from the analysis. When it was possible to deduce the identity of singers and the duration of songs from the remaining channels, the data were annotated.

Sound files typically lasted ~2–4 h, interspersed by silent periods of ~1 min. Song histograms were generated by aligning songs from a canary to the song onsets of a reference canary centered at time = 0s, and summing the number of songs at each time bin. Songs contributed to the song histogram for their entire duration (Figure 4). Alternatively, in histograms of song onsets, only the first bin contributed to histograms (Supplementary Figure S1). To construct histograms, we excluded the songs during the first and the last 60 s of each recording. Histograms were generated individually for each recording period. Histograms for all recording periods were then summed to generate the histograms for all days.

Histograms were also generated after randomization of songs from the analyzed bird during the recording period. Randomizations were performed according to the “KeepGaps” method (Masco et al., 2016). In this randomization method, song durations and silent intervals durations between songs were shuffled to preserve both song and silent period statistics, ensuring that statistics of canary songs, which typically occur in song bouts, are preserved. Randomizations were performed for each corresponding recording period. Given that we excluded the first and last 60 s for the alignment of songs before generating histograms, if the randomized song distributions resulted in songs within these periods, a new randomization of songs in the recording period was generated. The mean randomized song histogram and 95% confidence interval were calculated from 20 randomizations.

Occasionally, we recorded periods of complex interactions in which birds repeatedly overlapped each other, and in rare interactions, the three birds overlapped their songs. Since these songs could be considered as both leading and following songs, and could therefore not be ascribed to clearly defined leader or follower categories, they were excluded from the analysis based on the two categories “Leader” and “Follower” for song duration analysis and end time differences for the two main duelists (Figures 6, 7).

One of the six recorded groups of birds was excluded from the analysis due to the low number of songs, which did not allow us to perform statistical analysis on singing interactions.

The songs recorded in the field were high-pass filtered for display using Audacity software, with a cutoff frequency of 2 kHz and a roll-off of 12 dB per octave.

### Statistical analysis

Data presented in the manuscript are expressed as mean ± S.E.M, unless otherwise stated. Statistical analyses were performed using R version 4.4.1 (R Core Team, 2021) and Igor Pro Software (version 8.04). The statistical tests used are specified throughout the manuscript.

The linear mixed-effects models were fitted using the package “lme4” in R. The models were fitted by restricted maximum likelihood (REML) estimation, and the *t*-tests for fixed effects were computed using Satterthwaite’s method, and implemented in the “lmerTest” package. The models performed are specified below:

In the first linear mixed-effects model, we tested the effect of song type on song duration. The response variable was the logarithm of song duration, and the independent categorical variable was song type, consisting of four categories: “Solo,” “Leader,” “Follower,” and “Following a song and then leading the next song.” Random effects considered were bird identity and group. Since including both group and bird as random effects resulted in singular fits, we considered two independent models, with the group, or alternatively, with bird identity as a random effect. Both models exhibited similar significance and estimates of fixed effects. We selected the model with bird identity as a random effect, as it explained more variance with the random effect (0.00811 vs. 0.005555), had lower residual variance (0.06567 vs. 0.070506), and yielded a lower REML criterium at convergence (792.3 vs. 1204.4).

In the second linear mixed-effects model, we tested the effect of leader song duration on the end-time differences. The response variable was the end-time differences between follower and leader songs, and the independent variable was the logarithm of leader song duration. The difference in end times between following and leading songs was calculated as the difference between the end times of the follower and the leader song. The random effects were bird identity and group.

In the third linear mixed-effects model, we tested the effect of follower song duration on leader song duration. The response variable was the logarithm of the leader song duration, the independent variable was the logarithm of the follower song duration, and the random effects were group and leader identity.

## Data Availability

The original contributions presented in the study are included in the article. Further inquiries can be directed to the corresponding author.
