# Supplementary material for: Telemetry reveals rapid duel-driven canary song plasticity in a competitive social environment
Source: Front Psychol. 2025 Mar 5;15:1468782. doi: 10.3389/fpsyg.2024.1468782 (PMC11927533; doi:10.3389/fpsyg.2024.1468782)
Supplement: Supplementary file 1 [file Data_Sheet_1.pdf]

Group	Bird	Total solo	Total overlap	Total all	Ratio overlap/ total bird	Ratio bird overlap/ total group overlap	Days recorded
1	Bird 1 (Duelist 1)	100	73	173	0.422	0.474	7
1	Bird 2 (Duelist 2)	41	81	122	0.664	0.526	7
1	Bird 3	157	0	157	0.000	0.000	7
2	Bird 1 (Duelist 1)	751	208	959	0.217	0.486	12
2	Bird 2 (Duelist 2)	92	203	295	0.688	0.474	12
2	Bird 3	361	17	378	0.045	0.040	12
3	Bird 1 (Duelist 1)	236	281	517	0.544	0.549	10
3	Bird 2 (Duelist 2)	155	219	374	0.586	0.428	10
3	Bird 3	94	12	106	0.113	0.023	10
4	Bird 1 (Duelist 1)	308	394	702	0.561	0.429	8
4	Bird 2 (Duelist 2)	354	524	878	0.597	0.571	8
4	Bird 3	2	0	2	0.000	0.000	8
5	Bird 1 (Duelist 1)	428	256	684	0.374	0.415	8
5	Bird 2 (Duelist 2)	497	332	829	0.400	0.538	8
5	Bird 3	53	29	82	0.354	0.047	8

Supplementary Table 1. Summary of total, solo, and overlapping songs and the number of days recorded.

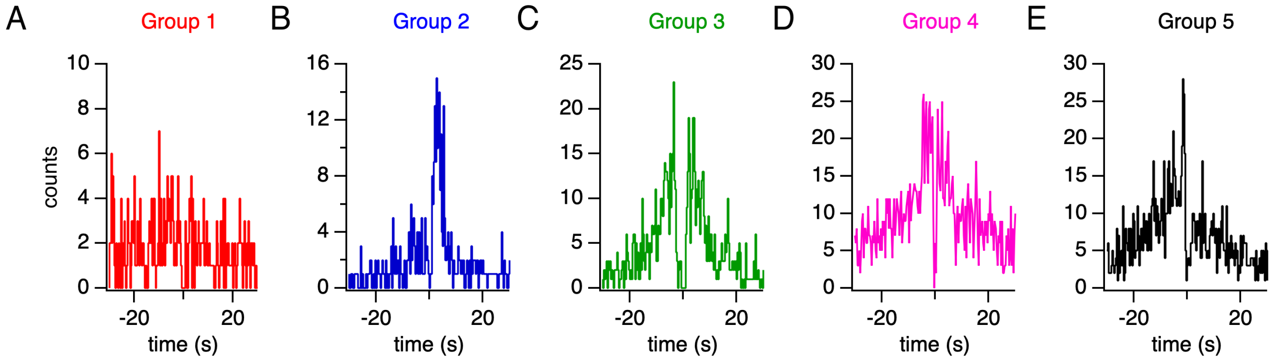

Supplementary Figure 1. Histograms of song onsets of duelist 1 relative to songs of duelist 2 aligned at song onset. (A-E) Data for each group.

		fight	no fight	Fisher's exact test		Proportion of fights overlap	solo
Group1	overlap	8	7			0.533	0.133
	solo	6	39	p = 0.0034			
Group2		fight	no fight				
	overlap	4	9			0.308	0.029
	solo	1	33	p = 0.0167			
Group3		fight	no fight				
	overlap	15	6			0.714	0.231
	solo	3	10	p = 0.0122			
					Average	0.518	0.131
					S.E.M.	0.117	0.058

Supplementary Table 2. Statistics of fights following solo and overlapping songs.

		Solo-Leader	Solo-Follower	Leader-Follower
Group1	Bird1	1.525433e-09	1.267095e-03	1.611660e-02
	Bird2	7.893767e-05	1.495229e-01	1.351101e-06
Group2	Bird1	2.902076e-19	3.281867e-06	2.728541e-03
	Bird2	3.384616e-05	4.410342e-03	4.118756e-07
Group3	Bird1	3.532779e-13	1.015553e-01	9.920268e-12
	Bird2	3.963186e-10	1.114358e-09	3.492753e-25
Group4	Bird1	7.647170e-08	9.265510e-03	7.201244e-11
	Bird2	1.262799e-08	7.272585e-12	1.140005e-29
Group5	Bird1	1.611949e-15	3.639751e-01	5.007314e-05
	Bird2	0.02425007	0.26368863	0.02085943

Supplementary Table 3. P-values for pairwise comparisons of song durations for solo, leading and following songs for the two main duelists in each group, related to Figure 6 A1-A5. Statistical analyses performed using Dunn's test with Bonferroni-Holm correction.
